# Targeting TL1A/DR3 Signaling Offers a Therapeutic Advantage to Neutralizing IL13/IL4Rα in Muco-Secretory Fibrotic Disorders

**DOI:** 10.3389/fimmu.2021.692127

**Published:** 2021-07-08

**Authors:** Hope Steele, Kacey Sachen, Andrew J. McKnight, Rachel Soloff, Rana Herro

**Affiliations:** ^1^ Division of Immunobiology, Cincinnati Children’s Hospital Medical Center, Cincinnati, OH, United States; ^2^ Kyowa Kirin Pharmaceutical Research, Inc., La Jolla, CA, United States; ^3^ Department of Pediatrics, University of Cincinnati, Cincinnati, OH, United States

**Keywords:** mucus, asthma, TL1A, IL13, fibrosis

## Abstract

Mucus secretion is an important feature of asthma that highly correlates with morbidity. Current therapies, including administration of mucolytics and anti-inflammatory drugs, show limited effectiveness and durability, underscoring the need for novel effective and longer lasting therapeutic approaches. Here we show that mucus production in the lungs is regulated by the TNF superfamily member 15 (TL1A) acting through the mucus–inducing cytokine IL-13. TL1A induces IL13 expression by innate lymphoid cells leading to mucus production, in addition to promoting airway inflammation and fibrosis. Reciprocally, neutralization of IL13 signaling through its receptor (IL4Rα), completely reverses TL1A-induced mucus secretion, while maintaining airway inflammation and fibrosis. Importance of TL1A is further demonstrated using a preclinical asthma model induced by chronic house dust mite exposure where TL1A neutralization by genetic deletion or antagonistic blockade of its receptor DR3 protected against mucus production and fibrosis. Thus, TL1A presents a promising therapeutic target that out benefits IL13 in reversing mucus production, airway inflammation and fibrosis, cardinal features of severe asthma in humans.

## Introduction

Many fibro-proliferative disorders of the lung such as asthma and chronic obstructive pulmonary disease (COPD) exhibit increased morbidity associated with mucus hypersecretion (1, 2). Existing therapies are available, but these have varying effectiveness. Bronchodilators do not specifically target mucus hypersecretion (3, 4). Moreover, severe asthmatics become gradually resistant to corticosteroids (5–7). Therefore, there is an absolute necessity to develop novel therapies to limit mucus hypersecretion and improve patient outcomes.

Interleukin-13 (IL13) has recently emerged as a primary target for asthma therapy, with ongoing clinical trials targeting IL13 or approved therapeutics targeting its receptor interleukin-4 receptor α (IL4Rα) (8, 9). It is thought that IL13 is central for mucus hypersecretion *via* direct effects on bronchial epithelial cells, in addition to promoting smooth muscle contractility, inflammation and fibrosis (10, 11). *In vitro*, lung epithelial cells that were differentiated in an air-liquid interphase and stimulated with IL13, increased their expression of ClcA1, a goblet cell-derived calcium-activated chloride channel tightly linked to mucus production, in addition to the mucin MUC5AC (10). Silencing ClcA1 inhibited IL13-driven mucin production by these cells, indicating that IL13 is dependent on ClcA1 to induce mucus production (10). IL4 another Th2 cytokine that binds to the IL4Rα, is unable to promote MUC5AC production by differentiated goblet cells, as compared to ones treated with IL13 (12). Despite their common receptor, IL4 and IL13 have non redundant activities in asthma. Whereas IL4 acts predominantly in the early phase of asthma development, IL13 is thought to be active in the late phase of allergic reactions. IL4 is involved in regulating T cell proliferation and survival, and IgE synthesis. In contrast, IL-13 is predominantly involved in airway remodeling and mucus hypersecretion (12–14). Several lines of evidence support the contention that IL13, and not IL4, controls mucus production in asthma. In fact, the *in vivo* blockade of IL13 alone, by genetic deletion (15) or antagonistic administration of the IL13Ra2-Ig both prevents and reverses established mucus cell changes (16). Anti-IL4 administration did not reduce the numbers of mucin-secreting cells in the bronchial epithelium of mice induced with ovalbumin (15). Moreover, the *in vivo* administration of recombinant IL13 in the airways enhanced mucus secretion, in IL4-deficient mice (17). Additionally, IL4-deficient Th2 cells adoptively transferred to mice receiving inhaled ovalbumin failed to enhance mucus production by their bronchial epithelium (18). Furthermore, conjugate vaccines against IL4 failed to reduce mucus production in murine model of asthma induced by HDM, as opposed to ones directed against IL13 that abrogated mucus production in the lung bronchial epithelium (14). Collectively this data indicates IL4 is unable to mount a muco-secretory phenotype independently of IL13.

Other soluble molecules could possess similar activities on goblet cells. We have recently discovered that TL1A is able to induce lung fibrosis and tissue remodeling associated with eosinophilic asthma and idiopathic pulmonary fibrosis (IPF) (19). TL1A (aka TNFSF15) is a member of the TNF superfamily that signals through death receptor 3 (DR3) (20, 21). We demonstrated that interrupting TL1A/DR3 signaling, by genetic deletion of the receptor or antagonistic blocking, decreases collagen deposition and smooth muscle accumulation in the lungs in two different models: the allergen-induced eosinophilic asthma model (Th2 asthma) and the bleomycin-induced IPF model (19). Airway resistance in response to methacholine was significantly decreased when active TL1A signaling was lacking (19). Moreover, we showed that the airway administration of recombinant TL1A in isolation is sufficient to promote collagen deposition and smooth muscle hypertrophy. We demonstrated that aside from its pro-inflammatory role, TL1A can exert direct activity on stromal cells (fibroblasts and epithelial cells) to induce fibrosis (19). This was an unprecedented discovery linking TL1A to tissue remodeling and lung fibrosis. Here, we investigated the role of TL1A in mucus production. This is interesting, as mucus plugs and airway obstruction lead to respiratory failure and constitute the main causes of death in asthma and COPD (22, 23).

In this work, we demonstrate that the intra-tracheal administration of TL1A into the airways induces mucus production (aside from fibrosis), and conversely, when blocking TL1A signaling through DR3, mucus production was abrogated in murine eosinophilic asthma. Additionally, we show that TL1A muco-secretory activity is indirect on goblet cells. TL1A induces IL13 production by group 2 innate lymphoid cells (ILC2), which subsequently promotes mucus production. TL1A represents a promising therapeutic target for muco-secretory diseases. This work has tremendous potential significance in human asthma, COPD and cystic fibrosis therapies.

## Materials and Methods

### Mice

Six- to eight-week-old male and female DR3-deficient mice and WT littermates (house on the C57BL/6 x 129 background), derived by Taconic Biosciences (#TF3529; Rensselaer, NY), were bred in-house. Male and female WT C57BL/6 or BALB/c and IL4Rα-deficient mice were purchased from Jackson Laboratories (Bar Harbor, ME). RAG2-deficient mice bred on the BALB/c x 129 background were a generous gift of Dr. Nunzio Bottini (UCSD), and RAG2γc-deficient mice on the BALB/c x 129 background were purchased from Jackson Laboratories (#014593). In all studies, both male and female mice were used. All studies and protocols were approved by and in compliance with the regulations of the Institutional Animal Care and Use Committees of Cincinnati Children’s Hospital Medical Center.

### Experimental Protocols

Activity of recombinant protein: WT C57BL/6 mice, RAG2-deficient mice, and RAG2γc-deficient mice, were given 10 μg of recombinant mouse TL1A (R&D Systems, Minneapolis, MN) or PBS intratracheally on days 1 and 2 and sacrificed for analyses one day later on day 3. Ocean Ridge Biosciences (Deerfield Beach, FL) performed the RNAseq transcriptomic screen and analysis on the lungs of mice induced with TL1A, *via* Illumina HiSeq 2000 (NCBI SRA database BioProject # PRJNA735223).

Asthma model: WT littermates and DR3-deficient mice on the C57BL/6 x 129 background, from both sexes, were sensitized i.n. on day 0, 7, and 14 with 200 and 100 μg house dust mite extract protein (HDM; GREER Labs Inc, Lenoir, North Carolina; endotoxin levels at 930EU/vial) in PBS, followed by chronic i.n. challenges of 50 μg of HDM protein administered twice a week for the following 4 weeks as previously described (24). Analyses were performed 24 hours after the last challenge. For neutralization of TL1A-DR3 interactions, mouse DR3-Fc (generous gift of Dr. Soloff) or isotype control IgG (BioXCell, Lebanon, NH) were administered intraperitoneal (i.p.) to WT C57BL/6 mice after the initial sensitization period starting at day 14 and were given every three days until the end of the experiment (100 μg/injection/mouse).

### Immunofluorescence Staining of ClcA1, MUC5AC, and α-Smooth Muscle Actin

Paraffin-embedded tissues are sliced 4μm thick each section. Deparaffinization is performed by successive incubation in xylene, followed by 100% ethanol and 75% ethanol for 5 min each time. Sections are washed in PBS before being treated with antigen retrieval solution (Citrate buffer pH 6) for 20 min in the microwave (Power 3) and rested for an additional 20 min at room temperature (RT). The sections are then washed in PBS and incubated in blocking solution containing 10% Donkey Serum and Fc block for 1h at RT. Primary antibodies for ClcA1 (clone EPR12254; Abcam, Cambridge, MA) and MUC5AC (clone 45M1; Abcam) are used at 1:100 dilution according to the manufacturer recommendations and incubation is performed overnight at 4°C. Sections are washed in PBS three times before staining with secondary antibodies Donkey anti-mouse and Donkey anti-rabbit (ImmunoJackson Research, West Grove, PA) at 1:500 dilution for 3h at RT. Sections are then washed in PBS three times and nuclear staining is performed using DAPI at 1:10000 dilution for 5 min at RT. Sections are mounted in ProLong Gold Antifade Mountant (Thermo Fisher Scientific, Waltham, MA) and imaged on Nikon Inverted immunofluorescence microscope.

### Immunohistochemistry Staining

PAS, Hematoxylin and Eosin (H&E) and Trichrome stains are performed according to the manufacturers’ recommendations (Poly Scientific R&D, Bay Shore, NY and Thermo Fisher Scientific). Sections are scanned using the Zeiss Axioscanner ×20 objective and analyzed using Image-Pro Premier software.

### Type 2 Innate Lymphoid Cells Sorting and DR3 Stain

Lung ILC2 are sorted on the following markers CD45.2^+^Lin^-^ Thy1.2^+^ ST2^+^Sca1^+^All antibodies are purchased from Biolegend (San Diego, CA) with exception to ST2 (101001PE, MD Biosciences). DR3 stain is performed using the clone 4C12 from Biolegend.

### ILC2 Culture and Stimulation With TL1A

ILC2 are cultured for five days in the presence of 50ng/ml IL2, IL7, and IL33 (Peprotech, Rocky Hill, NJ and R&D Systems) followed by two days culture with 10ng/ml IL2, IL7, and IL33 and 100 ng/ml TL1A (R&D Systems# 1896 TL). Measurements of IL13 in the culture supernatants is performed using mouse IL13 DuoSet ELISA (R&D Systems).

### Flow Cytometry

BAL and lung cells were treated with RBC lysing buffer (Sigma). Lungs were dissociated using a Lung Dissociation Kit (Miltenyi Biotec) and Gentle MACS (Miltenyi Biotec). LIVE/DEAD cells were stained with Fixable Aqua Dead Cell Staining Kit (Thermo Fisher Scientific), and after Fc block with the 2.4G2 mAb (eBioscience), cells were stained with the following Abs: DR3-PE (4C12; BioLegend), SiglecF (E50-2440; BD Biosciences), CD11b (M1/70; BD Biosciences), CD11c (HL3; BD Biosciences), Ly6G (1A8; BioLegend), ST2-PE (101001PE, MD), CD90.2 (30-H12; BioLegend). For lineage markers for ILC2 staining, the following Abs were used: CD3-FITC (145-2C11; eBioscience), CD4-FITC (GK1.5; eBioscience), CD8- FITC (5H-10-1; BioLegend), CD19-FITC (1D3; BioLegend), NK1.1-FITC (PK136; BioLegend), CD11b-FITC (M1/70; BioLegend), CD11c-FITC (HL3; BD Biosciences), and Gr1-FITC (RB6-8C5; BioLegend). Flow cytometry analysis was performed on a Fortessa (BD Biosciences), and data were analyzed using FlowJo Software (version 10; FlowJo, Ashland, OR). Live CD45+ lung immune cells were separated into T cells (CD3+, CD90.2+), macrophages (CD11b+, CD11c+ SiglecF+), DCs (CD11c+ MHC class II+), neutrophils (GR1+, CD11b+ SigF-), eosinophils (Ly6C+, SigF+, CD11c-) and ILC2 (Lin^-^ Thy1.2^+^ ST2^+^Sca1^+^).

### Statistical Analyses

Statistical analysis was performed using GraphPad Prism software. One way ANOVA or non-parametric Mann-Whitney U test was used where indicated. When One way ANOVA was used, multiple comparison was employed. A *P* value < 0.05 was considered statistically significant. All data are representative of at least three independent experiments with multiple mice as indicated or different donor cell populations.

## Results

### Intratracheal TL1A Induces a Muco-Secretory Signature in the Lung

**Figure 1:**
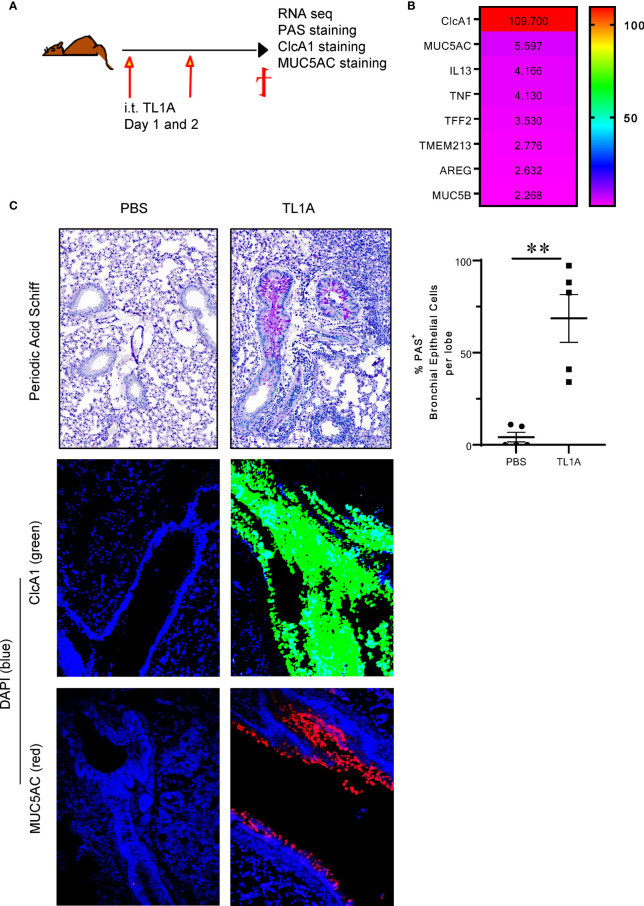
TL1A induces mucus production when administered in isolation into the airways. **(A)** Schematic representation of the protocol used. Briefly, C57BL/6 mice were induced with 10μg of recombinant TL1A i.t. on two successive days. Mice were euthanized 24h after the last i.t. injection, and lungs were harvested for RNAseq analysis, PAS, ClcA1 and MUC5AC stains. **(B)** Heatmap of the top differentially regulated genes involved in mucus production, upregulated in the lungs of TL1A-induced mice. **(C)** Top panel: PAS stain of mucus produced in the lungs and quantified using Image Pro Premier and graphed as %PAS+ bronchial epithelial cells per lobe (right panel). Mid and Lower panels: Immunofluorescence stains of CLcA1 (green), MUC5AC (red) and nuclear DAPI (blue). All results representative of three experiments with four to six mice per group. **p < 0.005.

We conducted a transcriptomic RNAseq analysis on murine lungs induced with recombinant TL1A, challenged on two successive days and euthanized 24h after the last injection (**Figure 1A**). We discovered that the highest differentially expressed gene in response to TL1A was chloride channel accessory 1 encoding the goblet cell protein ClcA1 (109.700 fold increase), which is involved in mucin synthesis (**Figure 1B**). ClcA1 is thought to signal through TMEM213 to promote MUC5AC production by bronchial epithelial cells stimulated with IL13 (10). In addition to ClcA1, many muco-secretory genes were upregulated in response to TL1A stimulation. These include genes encoding for IL13 (4.166 fold increase), the mucins MUC5AC (5.590 fold increase) and MUC5B (2.268 fold increase), TNFα (4.130 fold increase), TFF2 (3.530 fold increase), TMEM213 (2.776 fold increase) and AREG (2.632 fold increase) (**Figure 1B**). IL4 transcripts were not increased in the lungs of TL1A-induced mice. The intratracheal administration of TL1A in isolation to the airways induces a muco-secretory signature in the lung. Additionally, airway inflammation was increased in TL1A-induced mice compared to PBS (**Supplementary Figures 1A, B**). Flow cytometry analyses of immune cells performed on lung tissue showed a significant increase in T cells, eosinophils, neutrophils, macrophages and innate lymphoid cells (**Supplementary Figure 1A**). Consistent with the flow analysis, H&E scoring of lung biopsies demonstrated a significant increase in airway inflammation (**Supplementary Figure 1B**). We have additionally monitored fibrosis by scoring trichrome stain of collagen in lung biopsies and showed TL1A-induced mice increased airway fibrosis [**Supplementary Figure 1B** and (19)]. TL1A was able to induce changes in airway resistance and lung function [**Supplementary Figure 1C** and (19)]. Lastly, we monitored levels of IL4, IL5, IL13, TSLP and Periostin transcripts in the lungs of TL1A-induced mice by qPCR (**Supplementary Figure 1D**) and demonstrated mRNA transcripts of IL13 are the most highly increased in TL1A-induced mice (**Supplementary Figure 1D**). IL4 was not enhanced after the intratracheal instillation of TL1A in the airways (**Supplementary Figure 1D**).

After injecting recombinant TL1A directly into the airways, we assessed mucus production by Periodic Acid-Schiff (PAS) stain as well as ClcA1 and MUC5AC expression by immunofluorescence. We observed that the airway administration of 10μg TL1A in isolation into the airways on two successive days, was sufficient to promote mucus production 24h after the last injection (**Figure 1C**).

### Interrupting TL1A/DR3 Signaling Decreases Mucus Production in Allergen-Induced Asthma

**Figure 2:**
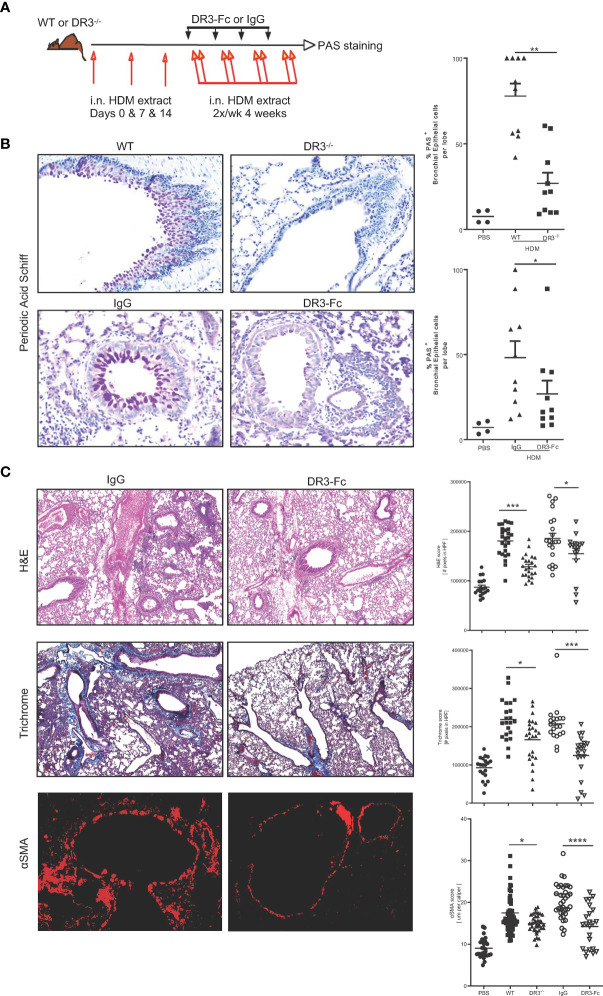
Interrupting TL1A/DR3 signaling decreases mucus production in allergen-induced asthma. **(A)** Schematic representation of protocol used. Briefly, WT littermates and DR3-deficient mice on the C57BL/6 x 129 background were sensitized i.n. on day 0, 7, and 14 with 200 and 100 μg house dust mite extract protein in PBS, followed by chronic i.n. challenges of 50 μg of HDM protein administered twice a week for the following four weeks. Analyses were performed 24 hours after the last challenge. For neutralization of TL1A-DR3 interactions, mouse DR3-Fc or isotype control IgG were administered i.p. to WT C57BL/6 mice after the initial sensitization period starting at day 14 and were given every three days until the end of the experiment (100 μg/injection/mouse). **(B)** PAS stain of mucus produced in the lungs and quantified using Image Pro Premier. **(C)** Inflammation assessed by H&E stain (top panels), collagen deposition assessed by trichrome stain (middle panels), and smooth muscle hypertrophy assessed by αSMA immunofluorescence stain (red) (bottom panels) on lung biopsies of WT C57BL/6 mice after treatment with either IgG or DR3-Fc. Quantifications for each parameter, completed using Image Pro Premier, are shown to the right, including untreated WT C57BL/6 and DR3^-/-^ mice (images not shown). All results representative of three experiments with five mice per group. *p < 0.05, ** < 0.005, ***p < 0.0005, ****p < 0.00005.

Chronic challenges with house dust mite in the airways after a sensitization phase, generates an asthma endotype (19, 24) with strong airway eosinophilia (24), Th2 (25) and type 2 innate lymphoid cells infiltrating the lung (26). This model involves type 2 cytokines like IL4, IL5, IL13 and periostin (19), IgE and alarmins like TSLP, IL25 and IL33 (27). We chronically induced wildtype and DR3^-/-^ mice with house dust mite (HDM from Greer Laboratories) for six weeks by first sensitizing the mice using 200μg HDM intranasal (i.n.) at day 0, 100μg HDM i.n. at day 7, and 50μg HDM i.n. at day 14. We then challenged the mice every other day with 50μg i.n. HDM for four weeks and monitored mucus production 24h after the last HDM dose (**Figure 2A**). Strikingly, mucus production assessed by PAS was significantly dampened in mice lacking an active TL1A/DR3 signal. Moreover, we treated wildtype mice induced with chronic HDM, with 100μg DR3-Fc, an antagonist reagent to TL1A, during the challenge phase (starting day 14) twice a week until the end of the experiment. Another group received similar doses of an isotype control to DR3-Fc. We showed that DR3-Fc significantly decreases mucus production, post-disease onset (**Figure 2B**). Additionally, inflammation and fibrosis, assessed by H&E, Trichrome and smooth muscle actin stains respectively, were significantly decreased when TL1A signal was removed by genetic deletion of its receptor DR3 or antagonistic neutralization (DR3-Fc treatment) (**Figure 2C**). Baseline trichrome and H&E scores were comparable between wild type and DR3-deficient mice (**Supplementary Figure 2A**). Flow cytometry analysis on bronchial lavage revealed strong airway eosinophilia compared to neutrophils which were modestly recruited. Additionally, cellular infiltrates were decreased in the bronchial lavage when TL1A signal was interrupted by genetic mutation of its receptor or antagonistic blockade, albeit not significantly (**Supplementary Figure 2B**). Strikingly, Lung ILC2 gated on CD45.2^+^Lin^-^ Thy1.2^+^ ST2^+^Sca1^+^ (**Supplementary Figure 2D**), were significantly decreased when TL1A signal was absent (**Supplementary Figure 2C**). Taken together, these data demonstrate that characteristic traits of asthma, including both mucus production and airway inflammation and remodeling, were drastically reversed post-TL1A neutralization.

### TL1A Muco-Secretory Activity Is Dependent on IL13/IL4Rα Signaling

**Figure 3:**
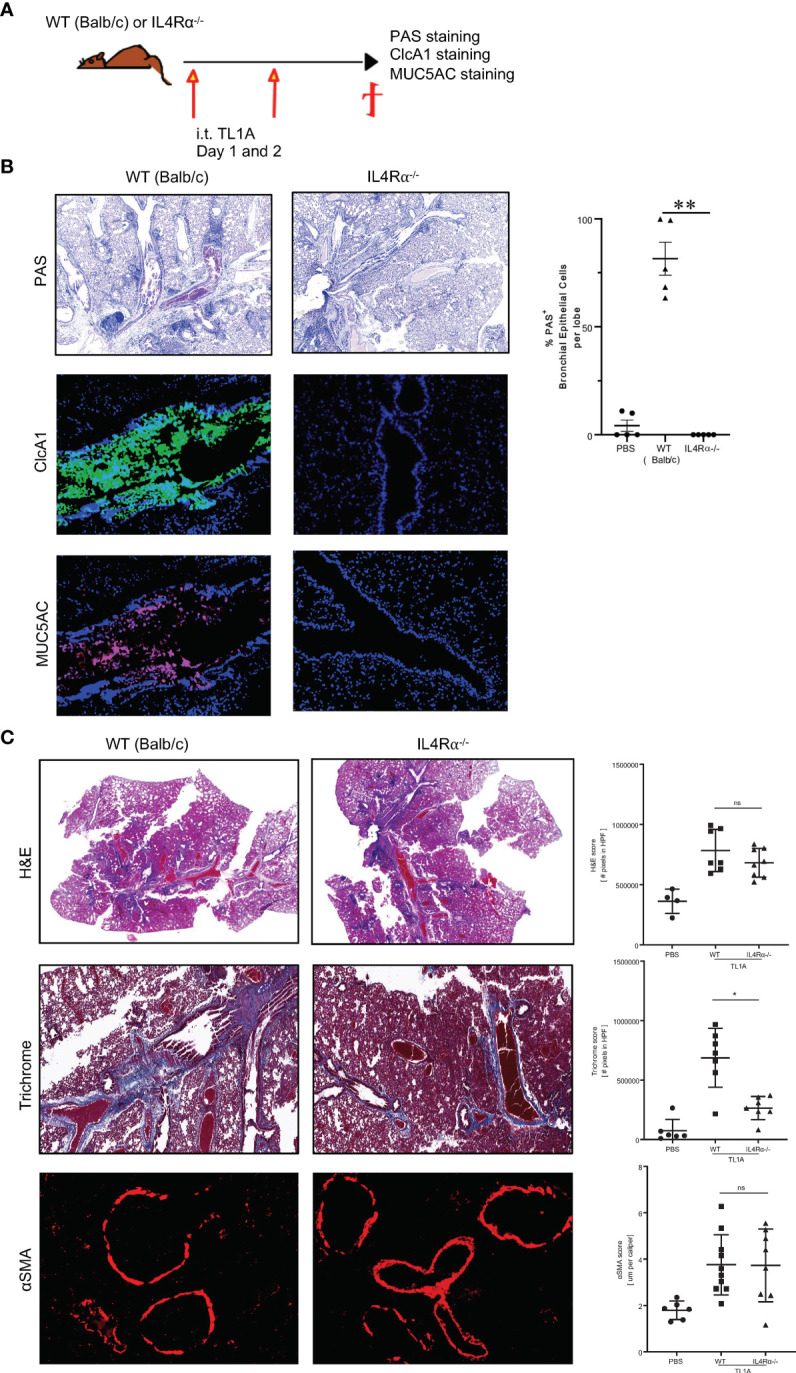
TL1A muco-secretory activity is dependent on IL13/IL4Rα signaling. **(A)** Schematic representation of the protocol used, as previously described in **Figure 1**. **(B)** Top panel: PAS stain of mucus produced in the lungs and quantified using Image Pro Premier (right panel). Mid and Lower panels: Immunofluorescence stains of CLcA1 (green), MUC5AC (red) and nuclear DAPI (blue). **(C)** Inflammation assessed by H&E stain (top panels), collagen deposition assessed by trichrome stain (middle panels), and smooth muscle hypertrophy assessed by αSMA immunofluorescence stain (red) (bottom panels) on lung biopsies of BALB/c and IL4Rα-deficient mice induced with PBS or TL1A. Quantification for each parameter is done using Image Pro Premier. All results representative of three experiments with four to six mice per group. ns, not significant, *p < 0.05, ** < 0.005.

IL13 is thought to play a central role in promoting mucus production associated with asthma and COPD, making IL13 neutralizing biologics the focus of both an approved therapeutic, dupilumab, and multiple other ongoing clinical trials (28–31). We questioned whether TL1A muco-secretory activity was dependent on IL13 signaling through its receptor IL4Rα, expressed on goblet cells. To answer this, we injected IL4Rα-deficient mice with recombinant TL1A on two successive days and monitored mucus production at day 3 (**Figure 3A**). Surprisingly, IL4Rα-deficient mice were completely mucus free as compared to wildtype BALB/c mice that induced mucus production after TL1A instillation (**Figure 3B**). TL1A muco-secretory activity is therefore completely dependent on IL13 signaling through its IL4Rα. Thus, we hypothesized that TL1A acts indirectly on goblet cells to promote mucus production by upregulating IL13. Indeed, IL13 transcripts were increased 21.3 folds in wildtype mice induced with TL1A and 17.7 folds in IL4Rα-deficient mice that can’t signal through IL13 despite the increase in its transcript expression (**Supplementary Figure 3B**). IL4 transcripts were not increased in neither the wildtype nor the IL4Rα-deficient mice (**Supplementary Figure 3B**). Most importantly, while mucus production was completely abrogated in IL4Rα-deficient mice, inflammation persisted (**Figure 3C**, top). Collagen deposition was lessened in IL4Rα-deficient mice (**Figure 3C**, middle), whereas smooth muscle hypertrophy remained unchanged (**Figure 3C**, bottom).

### TL1A Acts on Innate Lymphoid Cells to Promote IL13 Expression Necessary for Mucus Production, Independently of Adaptive Immunity

**Figure 4:**
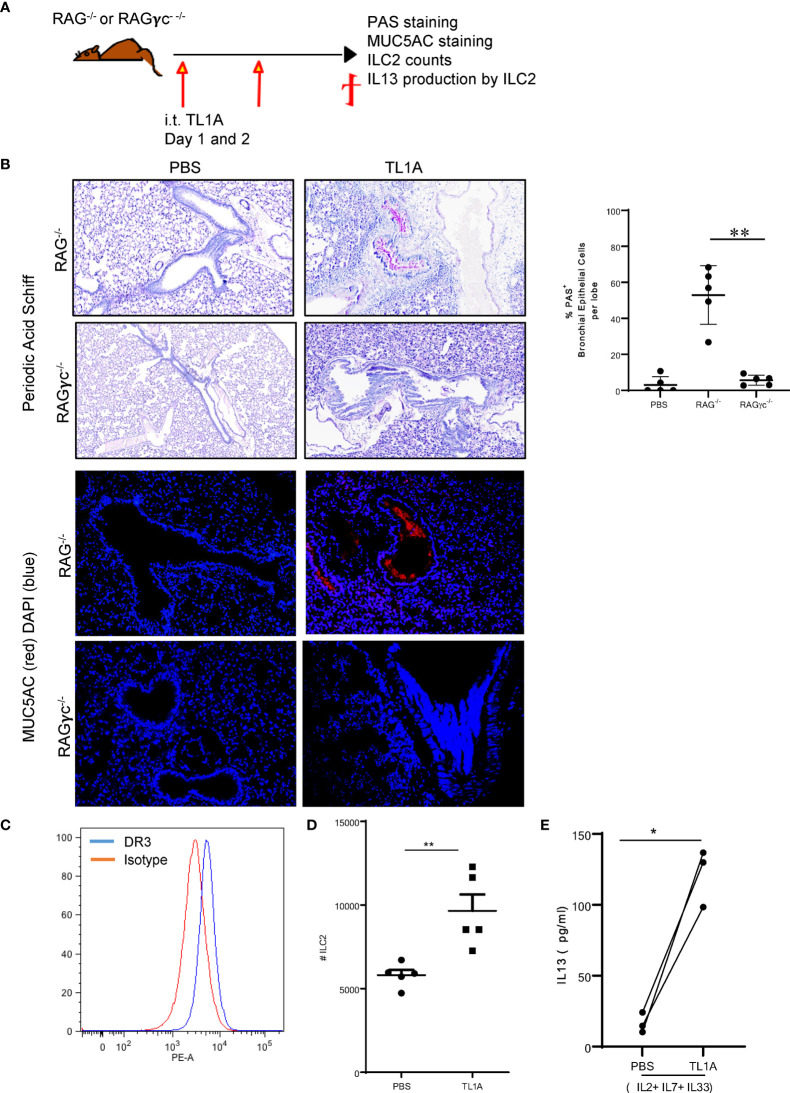
TL1A induces mucus production independently of adaptive immunity, bystander to IL13 production by ILC2. **(A)** Schematic representation of the protocol used, as previously described in **Figure 1**. **(B)** Top panel: PAS stain of mucus produced in the lungs of RAG^-/-^ and RAGγc^-/-^ mice induced with PBS or TL1A and quantified using Image Pro Premier (right graph). Lower panels: Immunofluorescence stains of MUC5AC (red) and nuclear DAPI (blue). **(C)** DR3 staining on ILC2 assessed by flow cytometry (DR3 in blue and Isotype in red). **(D)** Number of lung ILC2 in the left lobe of RAG^-/-^ mice induced with TL1A. **(E)** IL13 levels assessed by ELISA, in the supernatants of ILC2 cultured in the presence of IL2, IL7, and IL33 and stimulated with or without TL1A. All results representative of three experiments with four to six mice per group, or three individual replicate cultures. *p < 0.05, **p < 0.005.

The two predominant cellular sources of IL13 are T cells and innate lymphoid cells. We first questioned whether TL1A requires adaptive immunity, specifically, whether IL13-expressing T cells drive mucus production. For this purpose, we injected RAG-deficient mice with recombinant TL1A on two successive days and monitored mucus production (**Figure 4A**). Surprisingly, TL1A maintained its capacity to promote mucus production in mice lacking lymphocytes (**Figure 4B**). We therefore concluded that TL1A muco-secretory activity is independent of adaptive immunity.

Alternative to T cells, type 2 innate lymphoid cells (ILC2) are major cellular sources of IL13 and are increased in allergen-induced asthma (32, 33), but not IL4 (13, 34). We therefore questioned whether TL1A induces IL13 production by innate lymphoid cells to control mucus production. We injected 10μg of recombinant TL1A on two successive days into RAGγc-deficient mice that lack ILC in addition to lymphocytes (**Figure 4A**). TL1A was unable to induce mucus production in the RAGγc-deficient mice as compared to RAG-deficient mice (**Figure 4B**). Moreover, TL1A increased 14.8 folds IL13 transcripts in the lung of RAG-deficient mice, whereas TL1A-induced RAGγc-deficient failed to enhance IL13 expression (**Supplementary Figure 3A**). IL4 levels remained unchanged in both RAG and RAGγc-deficient mice, challenged with TL1A (**Supplementary Figure 3A**). This data suggests TL1A acts on ILC to promote IL13 production. To determine whether TL1A exerts a direct effect on ILC, we first verified they express its receptor DR3. We sorted lung ILC2 cells from the lungs of RAG-deficient mice induced with TL1A and examined DR3 expression. Lung ILC2 sorted on CD45.2^+^Lin^-^ Thy1.2^+^ ST2^+^Sca1^+^ expressed DR3 on their surface (**Figure 4C**). *In vivo*, TL1A induced proliferation and activation of lung ILC in RAG-deficient mice (**Figure 4D**). We observed an increase in the numbers of lung ILC2 (**Figure 4D**) as well as upregulation of IL13 production by these cells (**Figure 4E**). ILC2 cultured for five days in the presence of 50ng/ml IL2, IL7, and IL33 followed by two days culture with 10ng/ml IL2, IL7, and/IL33 and 100 ng/ml TL1A, increased IL13 secretion assessed by ELISA in the culture supernatant (**Figure 4E**). We concluded TL1A promotes IL13 production by ILC2, which is necessary for mucus secretion.

## Discussion

**Figure 5:**
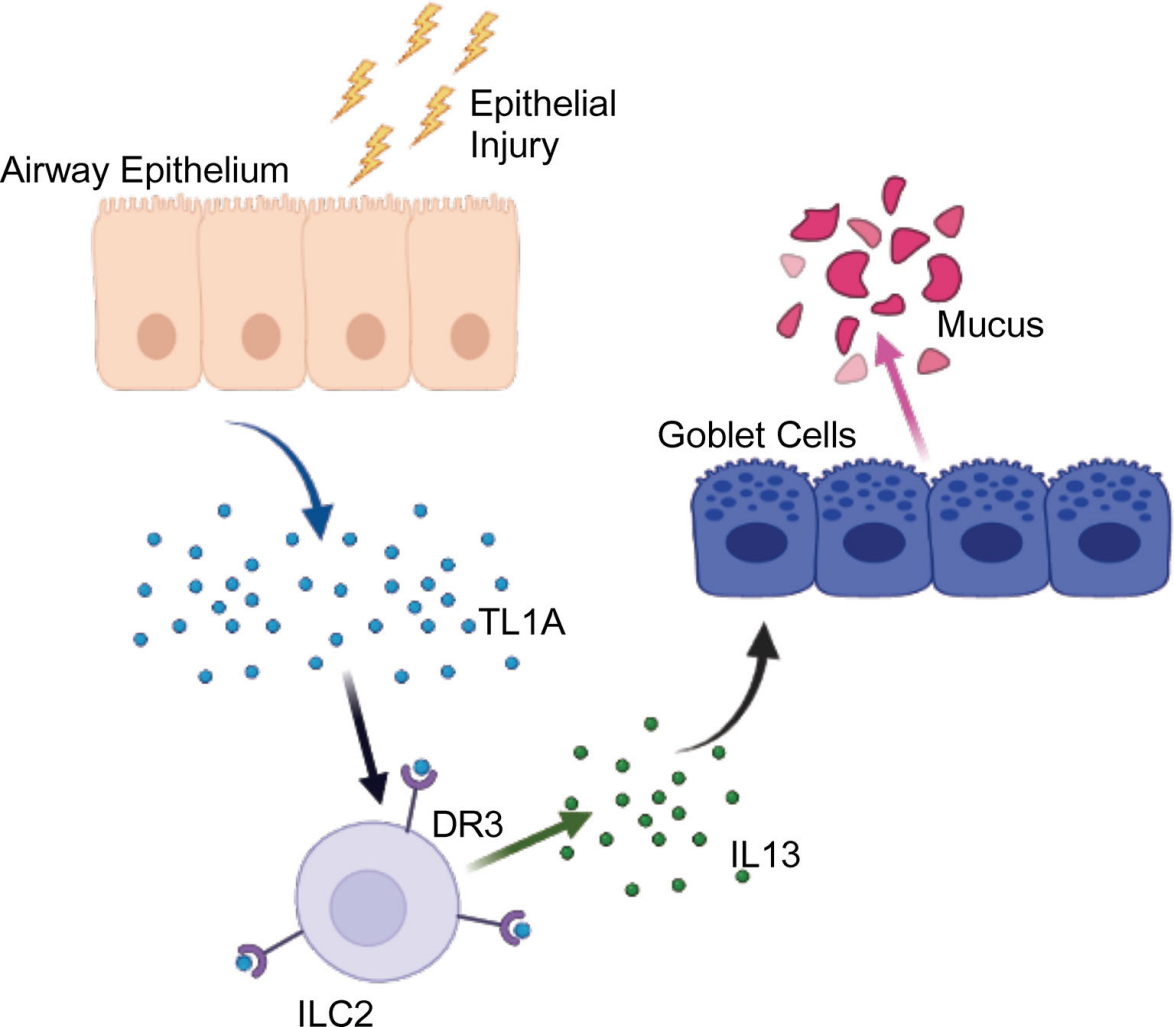
Graphical Abstract. Upon epithelial injury, such as exposure to allergens, TL1A is produced and acts on ILC2 *via* DR3 to promote IL13 secretion, leading to mucus production. Created with BioRender.com.

Mucus plugs are the major cause of death in asthma, COPD and cystic fibrosis. Current therapies involve the administration of mucolytics; however, these aren’t always effective at improving patient outcomes and there seems to be an increase in mortality associated with hypersecretory disorders. In this work, we describe, for the first time, a novel activity of TL1A as a driver of mucus production in the lung. To date, the most characterized cytokine involved in mucus production is IL13. It has been shown to drive mucus production by goblet cells differentiated *in vitro*, in Air-Liquid-Interface (ALI) (10). Additionally, silencing IL13 signaling through MAPK inhibition abrogated mucus production in murine models of asthma (35, 36). We have evidence that TL1A drives IL13 production by ILC2, which are increased in asthma. This is consistent with prior findings, showing that TL1A can promote allergic immunopathology through ILC2s (37, 38). Moreover, we show that TL1A can sustain a muco-secretory activity in the absence of T and B cells; however, ILC2 cells were necessary for this activity (**Figure 5**). ILC2 cells stimulated with TL1A, in the presence of the homeostatic cytokines IL2, IL7 and IL33, promoted IL13 production by these innate cells. We demonstrate that blocking TL1A signaling through its receptor DR3, by genetic deletion or antagonist blocking, abrogated mucus and IL13 production. Taken together with our data, which shows that blocking TL1A reduces collagen deposition and smooth muscle hypertrophy [(**Figure 2C** and (19)], targeting TL1A appears to be a novel approach to treat all features of airway inflammation and remodeling associated with asthma. TL1A acts upstream of IL13, driving mucus production through direct activity on ILC2. TL1A can act directly on structural cells of the lung, namely fibroblasts and epithelial cells, to promote airway remodeling and fibrosis.

Targeting TL1A shows a competitive advantage to neutralizing IL13, as this work shows that IL4Rα-deficient mice sustained an inflammatory response and smooth muscle hypertrophy, despite the absence of mucus and subsequent to the lack of an active IL13 signal. In humans, eight clinical trials failed to demonstrate benefit in neutralizing IL13 in severe asthmatic patients (28, 39). Three monoclonal antibodies directed against IL13 (tralokinumab, lebrikizumab and anrukinzumab) were used to interrogate the role of this cytokine in the pathophysiology of severe asthma and responses to treatment. The primary end points included FEV_1_ measurements, Mean ACQ6 scores, and asthma exacerbations recorded by the intake of systemic glucocorticoids and number of hospitalizations (29). All three antibodies showed a pattern of improvement without reaching significant decrease in the endpoints measured. Alternative to anti-IL13 antibodies, IL4Rα antagonists (dupilumab) have been recently FDA approved as add-on-maintenance therapy to treat moderate-to-severe asthma (31). Add-on therapy with dupilumab significantly reduced the oral glucocorticoid dose while simultaneously reducing the rate of severe exacerbations and improving lung function (FEV_1_) in patients with glucocorticoid-dependent severe asthma (31). More recently, in the “Liberty Asthma Quest Trial,” dupilumab treatment lowered significantly the rates of severe asthma exacerbation and improved lung function as compared to placebo treated controls. Greater benefits were seen in asthmatic patients with higher eosinophilia (31). Nevertheless, the effect of dupilumab on mixed granulocytic or neutrophilic asthma patients that are resistant to steroid treatment remain unanswered. It is becoming evident that neutralizing IL13 is not sufficient to reverse certain features of airway inflammation and fibrosis once established. Consistent with this, our study in the IL4Rα-deficient mice induced with TL1A demonstrated a complete abrogation in mucus production and reduced collagen deposition, while inflammation and fibrosis persisted. IL4 was not increased after TL1A airway challenge whereas IL13 was significantly induced. Additionally, several lines of evidence have refuted the idea that IL4 drives mucus production by bronchial epithelial cells. Interrupting IL13 in isolation by genetic deletion or antagonistic blockade reversed established mucus secretion (15, 16), whereas anti-IL4 blockade did not reduce mucin-secreting goblet cells in a murine model of asthma (15). Vice versa, IL13 administration in IL4-defiicent cells enhanced mucus production (17), supporting a unique role for IL13 in regulating mucus secretion. Collectively, this indicates that IL13, not IL4, controls mucus secretion through IL4Rα signaling.

Recently we showed soluble TL1A can be detected in the bronchial lavage of mice. TL1A is expressed on the surface of alveolar macrophages, dendritic cells, innate lymphoid type 2 cells, and subpopulations of lung structural cells. DR3 was found on CD4 T cells, innate lymphoid type 2 cells, macrophages, fibroblasts, and some epithelial cells and is expressed primarily by alveolar macrophages (19). In asthmatic patients presenting with the eosinophilic endotype, DR3 is upregulated in sputum ILC2 after allergen challenge or by IL2, IL33, and TSLP stimulation *in vitro*. DR3^+^ILC2 significantly increased IL5 expression upon TL1A stimulation (40). Combined with our data showing TL1A is upstream of IL13, this indicates that targeting TL1A signaling offers an advantage over targeting IL13/IL4Rα or IL5 signaling in isolation to treat mucus secretion associated with asthma.

Taken together, we demonstrate that interrupting TL1A/DR3 signaling abrogates mucus production associated with asthma, post-disease onset. We further show that TL1A acts on ILC2 to promote IL13 secretion leading to mucus production. Aside from its muco-secretory activity, TL1A acts directly on stromal and immune cells, perpetuating fibrosis and airway inflammation. While mucus secretion was dependent on IL13/IL4Rα signaling, in this work, we demonstrate that interrupting IL4Rα was not sufficient to decrease smooth muscle hypertrophy, fibrosis and airway inflammation. Targeting TL1A is therefore, more beneficial than targeting IL13/IL4Rα signaling in asthma and muco-secretory disorders.

## Data Availability Statement

The datasets presented in this study can be found in NCBI SRA database BioProject # PRJNA735223.

## Ethics Statement

The animal study was reviewed and approved by The Institutional Animal Care and Use Committee, Cincinnati Children’s Hospital Medical Center.

## Author Contributions

HS and RH performed experiments and analyzed data. KS, AM, and RS contributed reagents and expertise. RH directed the study and wrote the paper. All authors contributed to the article and approved the submitted version.

## Funding

This work was supported by funds from NIH grant U01 AI095542-08 MIST (Mucosal Immunology Study Team) to RH and Kyowa Kirin Pharmaceutical Research, Inc. (KKR).

## Conflict of Interest

Authors KS, AM, and RS were employed by Kyowa Kirin Pharmaceutical Research, Inc. The authors declare that this study received funding from Kyowa Kirin Pharmaceutical Research, Inc. Authors KS, AM, and RS of Kyowa Kirin Pharmaceutical Research, Inc participated in discussing results and editing the manuscript.

The remaining authors declare that the research was conducted in the absence of any commercial or financial relationships that could be construed as a potential conflict of interest.

## Supplementary Material

Supplementary Figure 1 TL1A induces airway inflammation and remodeling. **(A)** Flow cytometry analyses of cellular infiltration in the lungs of TL1A-induced mice as previously described. Total numbers of leucocytes, T cells, eosinophils, neutrophils, macrophages, and dendritic cells are shown. **(B)** Immunohistochemistry analyses of cellular infiltration assessed by H&E and collagen deposition assessed by Masson Trichrome. Quantifications are shown on the right panel. **(C)** Airway hyperresponsiveness (AHR) to increasing concentrations of methacholine of TL1A versus PBSinduced mice is shown. **(D)** Fold change in mRNA levels of IL4, IL5, IL13, TSLP and periostin in the lungs of TL1A over PBS-induced mice are shown. All results are representative of 5 to 10 mice per group. ****p < 0.00005.

Supplementary Figure 2 TL1A inhibition decreases the infiltration of ILC2 in the lungs and airway fibrosis. **(A)** Quantification of Masson Trichrome and H&E scores in the lungs of HDM-induced mice are shown. **(B)** Flow cytometry analysis of cellular infiltration in the bronchial lavage of mice induced with HDM as previously described. The grey dotted line indicates baseline infiltration. **(C)** Numbers of ILC2 in the left lobe of the lungs of mice induced with HDM. The grey dotted line indicates baseline infiltration. **(D)** Gating strategy of ILC2 cells used during the sorting. All results are representative of 10 mice per group. One-way Anova using a multi-comparison to WT HDM is used to calculate the p values. *p < 0.05, ***p < 0.0005.

Supplementary Figure 3 IL13 and IL4 expression in the lungs of TL1A -induced mice. **(A)** Relative expression of IL4 and IL13 in the lungs of RAG and RAGγc-deficient mice induced with TL1A as previously described. **(B)** Relative expression of IL4 and IL13 in the lungs of WT (BALBc) and IL4Rα- deficient mice induced with TL1A as previously described. All results are representative of 4 mice per group. *p < 0.05.

## Abbreviations

IPF, Idiopathic pulmonary fibrosis; TNF, Tumor necrosis factor; TL1A, TNF-like ligand 1A; DR3, Death Receptor 3; ILC, Innate lymphoid cells; COPD, Chronic obstructive pulmonary disease; IL13, Interleukin 13; IL4Rα: Interleukin 4 receptor α; TNFSF15, Tumor necrosis factor superfamily member 15; ClcA1, Chloride channel regulator, Calcium-Activated-1; PAS, Periodic Acid-Schiff; H&E, Hematoxylin and Eosin; i.t., Intratracheal; i.n., Intranasal; i.p., Intraperitoneal.
