# Supplementary figures and images for: Targeting TL1A/DR3 Signaling Offers a Therapeutic Advantage to Neutralizing IL13/IL4Rα in Muco-Secretory Fibrotic Disorders
Source: Front Immunol. 2021 Jul 8;12:692127. doi: 10.3389/fimmu.2021.692127 (PMC8299868; doi:10.3389/fimmu.2021.692127)

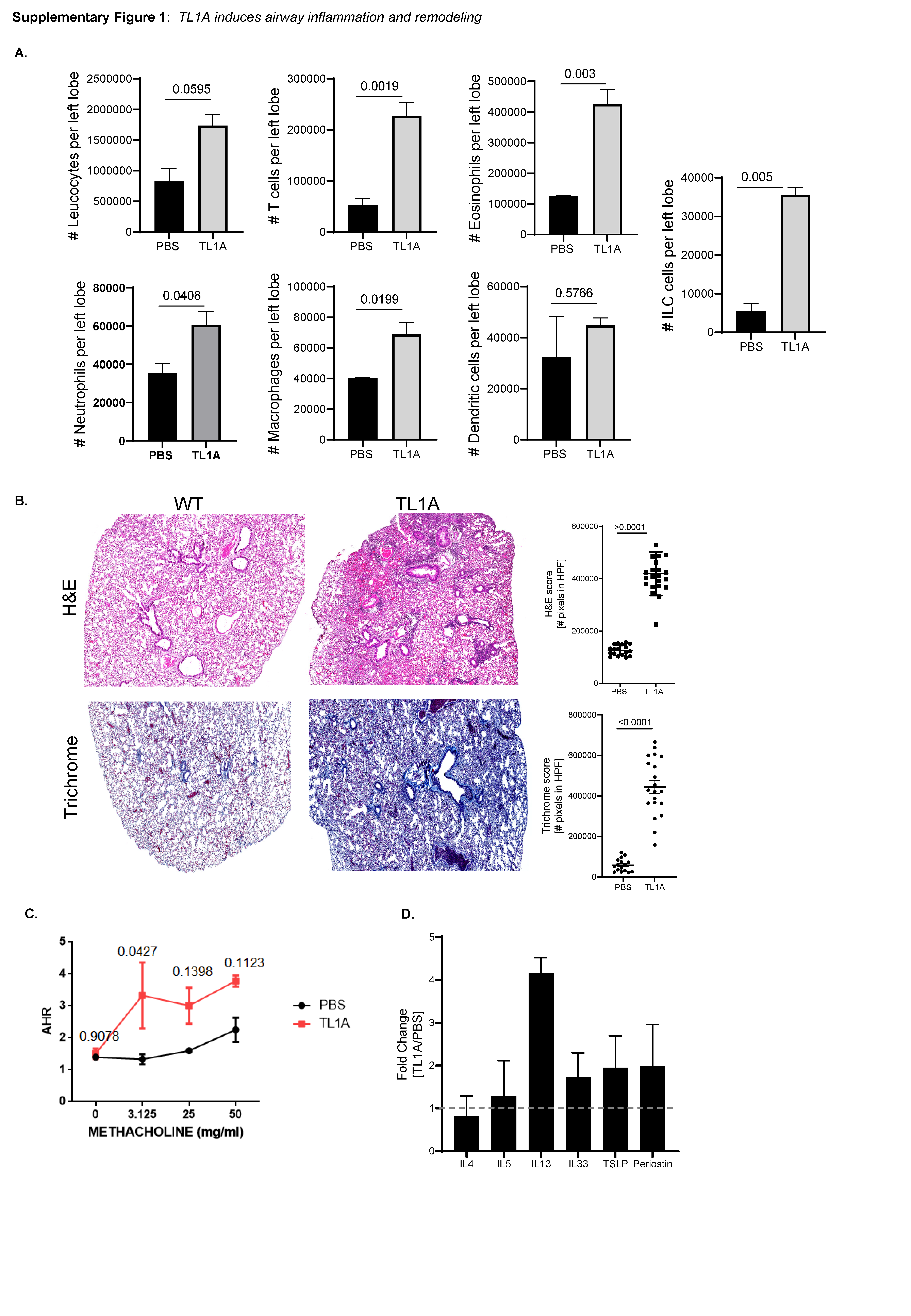

Supplement: Supplementary Figure 1 — TL1A induces airway inflammation and remodeling. (A) Flow cytometry analyses of cellular infiltration in the lungs of TL1A-induced mice as previously described. Total numbers of leucocytes, T cells, eosinophils, neutrophils, macrophages, and dendritic cells are shown. (B) Immunohistochemistry analyses of cellular infiltration assessed by H&E and collagen deposition assessed by Masson Trichrome. Quantifications are shown on the right panel. (C) Airway hyperresponsiveness (AHR) to increasing concentrations of methacholine of TL1A versus PBSinduced mice is shown. (D) Fold change in mRNA levels of IL4, IL5, IL13, TSLP and periostin in the lungs of TL1A over PBS-induced mice are shown. All results are representative of 5 to 10 mice per group. ****p < 0.00005. [file Image_1.tiff]
